# Grape Polyphenols in the Treatment of Human Skeletal Muscle Damage Due to Inflammation and Oxidative Stress during Obesity and Aging: Early Outcomes and Promises

**DOI:** 10.3390/molecules27196594

**Published:** 2022-10-05

**Authors:** Adriana Capozzi, Cédric Saucier, Catherine Bisbal, Karen Lambert

**Affiliations:** 1 PhyMedExp, INSERM U1046, CNRS UMR 9214, University of Montpellier, CEDEX 5, 34295 Montpellier, France; 2 SPO, INRAE, Institute Agro, University of Montpellier, 34000 Montpellier, France

**Keywords:** grape polyphenols, resveratrol, sarcopenia, skeletal muscle, clinical trial

## Abstract

Today, inactivity and high-calorie diets contribute to the development of obesity and premature aging. In addition, the population of elderly people is growing due to improvements in healthcare management. Obesity and aging are together key risk factors for non-communicable diseases associated with several co-morbidities and increased mortality, with a major impact on skeletal muscle defect and/or poor muscle mass quality. Skeletal muscles contribute to multiple body functions and play a vital role throughout the day, in all our activities. In our society, limiting skeletal muscle deterioration, frailty and dependence is not only a major public health challenge but also a major socio-economic issue. Specific diet supplementation with natural chemical compounds such as grape polyphenols had shown to play a relevant and direct role in regulating metabolic and molecular pathways involved in the prevention and treatment of obesity and aging and their related muscle comorbidities in cell culture and animal studies. However, clinical studies aiming to restore skeletal muscle mass and function with nutritional grape polyphenols supplementation are still very scarce. There is an urgent need for clinical studies to validate the very encouraging results observed in animal models.

## 1. Obesity and Aging: Two Major Healthcare Challenges to Solve

The population of elderly people is expanding worldwide with the older adults aged between 65–80 years being the fastest-growing portion, thanks to improvements in healthcare management which allows for increasing life expectancy [1]. Associated with this increase in lifespan, a global obesity epidemic is spreading due to life changes such as inactivity and high-calorie diets, favoring the growth of non-communicable diseases. Obesity and aging are together key risk factors for the development and progression of several chronic/non-communicable diseases (metabolic syndrome, Insulin Resistance (IR), Type 2 Diabetes (T2D) [2,3,4,5,6,7], sarcopenia [8,9], and frailty [10]. The World Health Organization (WHO) defines metabolic syndrome as a pathologic condition characterized by obesity (Body Mass Index (BMI) ≥30 kg/m^2^), IR, hypertension, and hyperlipidemia [11]). T2D is IR associated with decreased insulin secretion by the pancreas. [12]. Frailty is a clinical syndrome in elderly people comprising an increased risk for poor health outcomes, falls, incident disability, hospitalization, and mortality [13].

### 1.1. Skeletal Muscle Alterations Are Central 

Although non-communicable diseases associated with aging and obesity have different etiologies, development, and progression, they are all associated with skeletal muscle defect and/or poor muscle mass quality. Sarcopenia, defined as a loss of skeletal muscle mass and function [8], is frequently associated with aging and with a loss of independence, disability, frailty, and compromised quality of life, and, therefore, represents a high risk for morbidity and mortality [9]. Obese patients could also develop sarcopenia [7] and with the progression of obesity with aging [14], a growing number of obese sarcopenic patients is expected. Then, the management of skeletal muscle alteration during obesity and aging is mandatory.

Skeletal muscles are among the major tissues of the body, accounting for 40% of our total body weight and containing 50–75% of all body proteins. Moreover, skeletal muscles are responsible for more than 80% of glucose uptake after insulin stimulation, highlighting their central role in metabolism regulation. They ensure three main functions: posture and locomotion, thermoregulation, storage, and utilization of nutrients. Thus, they play a vital role in all our activities [15]. Growing evidence points to the central role of skeletal muscle in the systemic regulation of age-related diseases [16]. Indeed, functional and metabolic muscle alterations, as well as skeletal muscle mass decreasing, are all associated with the human mortality rate [17,18]. Then, impairment of skeletal muscle mass and/or function could lead to major pathologies, such as IR, T2D, and to weakness and disability which considerably decrease quality of life and are associated with a higher risk for morbidity and mortality [5,7,19,20].

### 1.2. Muscle Alterations

#### 1.2.1. Muscle Alterations in Obesity

**Figure 1:**
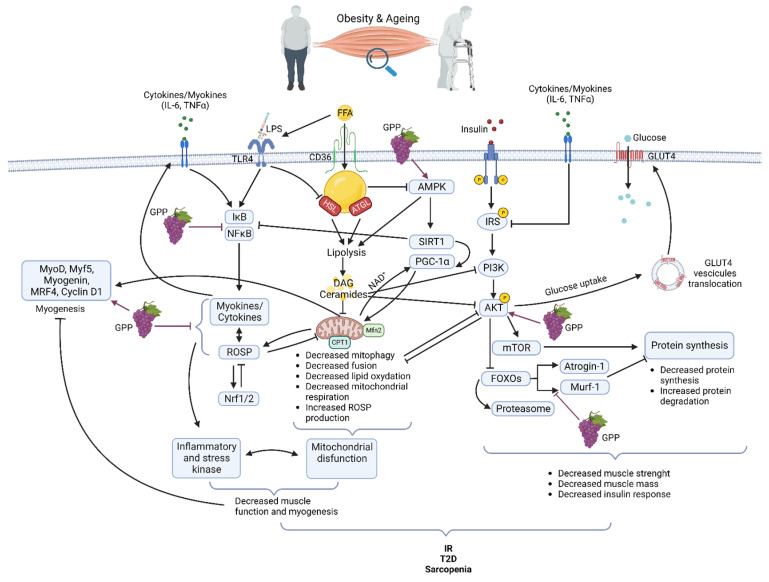
Main pathways involved in muscle dysfunction during obesity and aging and their modifications by grape polyphenols (GPP) (Created with BioRender.com).

Obesity exerts multiple effects on skeletal muscle metabolism. In obese grade I insulin-resistant women, only skeletal muscle insulin-signaling alteration was found with no variation in subcutaneous adipose tissue [21]. Moreover, the correlation of muscle alteration with glucose infusion rate during the hyperinsulinemic-euglycemic clamp underlines the major role of skeletal muscle in IR development [21]. Obesity is accompanied by increased ectopically lipid deposition in non-adipose tissues including skeletal muscle [22,23]. This lipid overload affects several cell signaling pathways and is associated with metabolic effects on mitochondrial function, insulin response, and energetic metabolism [24,25] (Figure 1). After their cell translocation by fatty acid translocase (FAT/CD36), a receptor and transporter for free fatty acid (FFA), lipids are stored as intramuscular triacylglycerols (TAG) in lipid droplets (IMTG). Myotubes from obese people present increased FFA uptake and esterification into complex lipids with overexpression of FAT/CD36 [23,26,27]. TAG turnover is also highly altered due to decreased lipolysis as lower hormone-sensitive lipase (HSL) and higher adipose triglyceride lipase (ATGL) protein levels are detected in the skeletal muscle of obese subjects [28]. Increased IMTG is also associated with higher levels of lipotoxic intermediates such as diacylglycerols (DAG) and ceramides. DAG might inhibit insulin signaling via the activation of the protein kinase C (PKC), which, in turn, decreases the activities of the phosphoinositide 3-kinase (PI3K) and of the insulin receptor substrate-1 (IRS1) in the insulin signaling pathway [29]. Ceramides have several functions in the alteration of skeletal muscle metabolism. They have been described as inhibitors of Akt phosphorylation via the activation of protein phosphatase 2A (PP2A) or, on the opposite, as inductors of Akt phosphorylation on an inhibitory residue via the activation of the protein kinase Cζ (PKCζ). Ceramides act also at the mitochondrial level by decreasing mitochondrial respiration, inhibiting oxidative phosphorylation, and promoting mitochondrial fragmentation. These activities lead to an induction of reactive oxidative species (ROSP) [30]. Decreased mitochondrial content in muscle of obese patients has also been attributed to impairment of mitochondrial biogenesis, decreasing the ability to oxidize lipids [31]. More recently, it was shown that mitochondrial lipid oxidation was impaired due to a decreased activity of the mitochondrial protein carnitine palmitoyltransferase 1 (CPT1). CPT1 is involved in the transport of long-chain fatty acids into the mitochondria. Then decreased CPT1 activity results in decreased β oxidation [32]. Circulating FFA could also induce a chronic low-grade inflammation through activation of toll-like receptor 4 (TLR4) and nuclear factor-kappa B (NFκB), resulting in the release of several pro-inflammatory cytokines as interleukines (IL) 6, 8, and 15 and tumor necrosis factor (TNF) α. These cytokines, as secreted by muscle, are also known as myokines. They could exert an autocrine or paracrine effect. However, it must be kept in mind that there is not a consensus on their secretion and their role in skeletal muscle inflammation and skeletal muscle metabolism dysregulation during obesity [21,33] Importantly, lipids overload also affects muscle maintenance and regeneration [34]. FFA accumulation decreases AMPKα activity. AMPKα promoted myogenesis by regulating the expression of miR-206 and miR-206’s target cyclin D1, which allows the regulation of the cell cycle and cell proliferation of muscular stem cells (satellite cells) during the skeletal muscle regeneration process [35]. By affecting general skeletal muscle function, mass, and quality, obesity reduces mobility [36] modifies lipid and carbohydrate metabolism and increases the risk of several comorbidities [37]. In fact, obesity accelerates the aging process and decreases life expectancy. Forty-year-old obese non-smoker females lost 7.1 years and forty-year-old obese non-smoker males lost 5.8 years of life expectancy [38].

#### 1.2.2. Muscle Alterations in Aging

The gradual decline of all physiological functions of all organs including skeletal muscle characterizes normal aging. During aging, due to an alteration of muscle quality, the loss of muscle strength precedes the loss of muscle mass [39,40]. However, muscle mass is lost as early as the fifth decade with acceleration from the age of 70 and sarcopenia is a hallmark of the aging process. Like in obesity, the reduction of muscle quality and mass with aging is the consequence of the interplay of a multitude of mechanisms [41] including damage caused by ROSP [42] inflammation [43], lipid infiltration [44], proteostasis imbalance [45] and mitochondrial impairment [46]. In aging like in obesity, alteration in mitochondria content, fusion, fission, and function are associated with muscle loss and muscle lipid accumulation [46] (Figure 1). Progressive mitochondrial dysfunction and ROSP accumulation are central to the aging process [47]. Aged skeletal muscle accumulates dysfunctional mitochondria due to a defect in mitophagy, mitochondrial biogenesis, and dynamics. Reduced expression levels of genes such as nuclear respiratory factor 1/2 (Nrf1/2), AMP-activated protein kinase (AMPK), peroxisome proliferative activated receptor gamma, coactivator 1 alpha (PGC-1α), mitofusin 1 and 2 (Mfn1/2) causes a reduction of mitochondrial number, mitochondrial content, mtDNA copy number, and impairment in mitochondria morphology in the skeletal muscle. Throughout life, the accumulation of dysfunctional mitochondria producing high ROSP levels contributes to the establishment of oxidative stress and increased risk of IR and T2D. Moreover, these defective mitochondria are unable to sustain enough energy in the cells, which results in progressive functional decline and cell death. [48,49,50]. Enhancement of ectopic fat deposit during aging is also accompanied by a resulting heightened production of pro-inflammatory cytokines (IL-6, TNFα) associated with lipotoxicity and leading to an increased risk of IR and T2D [51]. Protein quality control pathways, autophagy, and proteasome activity decrease participate also in the skeletal muscle dysfunctions and the decreased muscle mass observed during aging [45,52]. Maintaining muscle mass is a balance between protein synthesis and protein degradation systems. Aged skeletal muscle shows a marked defect in the contraction-induced activation of the protein synthesis pathway phosphatidylinositol 3-kinase/protein kinase B/mammalian target of rapamycin (PI3K/Akt/mTOR). Concerning the proteasome pathway and the muscle-specific ubiquitin ligase muscle RING-finger protein-1 (MuRF1) and atrogin-1 mRNA, levels in aged muscle are increased or unchanged [53,54].

The aging process presents several common mechanisms for obesity. On the other side, obesity in elderly people accelerates the aging process. Obesity and aging both deregulate cell metabolism and create a vicious circle that precipitates the aging process and the development of associated comorbidities [41,55]. Several mechanisms operating at different levels of muscle physiology are implicated in the muscle defects observed in obesity and aging. However, oxidative stress and inflammation are central components at the onset of muscle defect regardless of its etiology [6,19,56,57,58].

## 2. Oxidative Stress and Inflammation: Two Essential Harms 

Oxidative stress occurs when there is an imbalance between the production of ROSP in the cells and tissues and the antioxidant systems, which are responsible for their neutralization and removal [59]. Reactive oxidative species include derivatives of oxygen (Reactive Oxygen Species, ROS), nitrogen (Reactive Nitrogen Species, RNS), and sulfur (Reactive Sulfur Species, RSS), capable of oxidizing different substrates. The antioxidant defense system involves non-enzymatic scavengers provided by food as vitamins (trans retinol 2, vitamin A; ascorbic acid, vitamin C; α-tocopherol, vitamin E), carotenoids, polyphenols, and endogenous antioxidant enzymes superoxide dismutase (SOD), catalase, glutathione peroxidase (GPx), glutathione transferase (GST) (see [60,61] for complete reviews). 

If an excess of ROSP can be extremely deleterious, their regular generation is necessary for the physiological maintenance of all the tissues of our body and among them, the skeletal muscle [62]. In fact, via oxidation of redox-sensitive protein-cysteine, ROSP act as second messengers. They are important signaling molecules regulating metabolism, cell growth and differentiation, cell repair, immunity, and so on [59,63]. ROSP production is thus beneficial and essential to cell and tissue function. As oxidative stress, inflammation is also essential for normal organ function. Inflammation is a protective biological response aimed at identifying and eliminating a threat. Inflammation could be triggered by infection or not and participates in the activation of the immune system. It is the first line of defense against pathogens, but it also allows for repairing cell damage and tissue injury [64,65]. ROSP production and inflammation have dual roles. They are activated during several physiological responses, and they play an essential role in cellular signaling and regulatory pathways. However, they must be tightly regulated to avoid the development of oxidative stress, tissue injury, and chronic inflammation, which are detrimental to normal cells and tissues. In fact, when ROSP are produced in excess or inadequately they can cause irreversible damage to cells by oxidizing plasma membranes, DNA, proteins, and lipids [66]. In the same way, uncontrolled inflammation could lead to chronic inflammation and inflammatory diseases that could affect absolutely all the organs as skeletal muscle [64,67]. Interestingly, ROSP and inflammation could regulate each other in a two-way reciprocal direction [51]. Both ROSP production and inflammation participate together in skeletal muscle tissue repair [52]. However, during obesity and aging, ROSP production and inflammation are increased while antioxidant systems and anti-inflammatory pathways decreased in skeletal muscle [21,52,53]. In obesity and aging, high plasma levels of free fatty acids (FFA) and increased concentrations of lipopolysaccharide (LPS) from gut microbiota (due to increased permeability of gut), bind to Toll-like Receptor 4 (TLR4) [68,69]. TLR4 is an innate immunity receptor present in the skeletal muscle that can activate NFκB and inflammation via the MyD88 pathway. Moreover, the E3 ubiquitin ligase RNF41 has been found to participate in the TLR4 inflammation pathway in the muscle of insulin-resistant grade I obese women [56]. An excess of FFA is also responsible for deleterious effects on mitochondria such as uncoupling of oxidative phosphorylation, energy failure, decreased clearance, decreased fission, and release of ROSP [70]. Mitochondrial dysfunction and inflammation/oxidative stress together are responsible for a decrease in myogenesis and muscle function [71]. In skeletal muscle, elevated ROSP levels concurrently inhibit anabolic pathways as PI3K/Akt/mTOR [72], contributing to muscle mass loss and atrophy [73,74], and activating several mechanisms of the catabolic pathways (Figure 1). Under physiological conditions, Akt phosphorylates and inhibits the Forkhead box O transcription factors (FOXOs), thus inhibiting the muscle-specific ubiquitin ligase MuRF1 and atrogin-1, of the ubiquitin-proteasome system. With the inhibition of Akt, atrogin-1, MuRF-1 and proteasome are activated resulting in protein degradation [75]. Activation of the cysteine proteases, calpain, and caspase 3, which play a key role in the initial breakdown of sarcomeres during atrophic conditions, also participates in protein breakdown and muscle atrophy. The expression of inflammatory myokines such as tumor necrosis factor-alpha (TNFα) and interleukin 6 (IL6) is induced due to an increase in the activity of the transcription factor nuclear factor kappa B (NFκB) (Figure 1). NFκB activity can be increased by ROSP but also by several other stimuli such as free fatty acids (FFA), advanced glycation products, and inflammatory cytokines induced by oxidative stress [19]. On the other way, high levels of ROSP activate the nuclear factor, erythroid 2-like 2 (Nrf2) pathway leading to increased transcription of genes coding for antioxidant proteins, and consequently inducing the antioxidant ROSP-fighting effects [76]. 

## 3. Grape Polyphenols: An Effective Tool

Adapted diet and physical activity are known for several years as central countermeasures to avoid the deleterious physiological effects of obesity and aging on tissue homeostasis and to promote a healthy life. Treatment of obesity is difficult, and initially based on lifestyle change, diet recommendations, and increased physical activity [77] but, whereas it is actually effective, it is associated with very low compliance. Aging is inevitable but it is desirable to age in a healthy way, therefore the purpose of our society is now to increase the rate of healthy aging to avoid harmful consequences on skeletal muscle mass and function and to limit frailty and dependence. As stated above, oxidative stress and chronic inflammation are core mechanisms associated with obesity and aging. Among the numerous natural chemical compounds tested for their antioxidant and anti-inflammatory properties, grape polyphenols present great interest. 

Attention to the importance of dietary intake of polyphenols was ignited by the phenomenon called the ‘French Paradox’, first described by Serge Renaud from the University of Bordeaux in 1992. According to his observations, the French population when compared to other Western populations whose diet is rich in saturated fatty acids (e.g., the American population), shows a much lower incidence of coronary heart disease and associated mortality [78]. In fact, adherence to the Mediterranean diet which includes mainly plant-derived foods and red wine consumption has been associated with a lower risk of chronic diseases and mortality [79] and a lower frailty index in older adults [80]. The first explanation put forward to explain these associations was the moderate consumption of red wine. Undeniably, among fruits, grapes (but also red wine, grape seeds, and grape pomace) contain high amounts of polyphenols [81] (Table 1), although not being the richest source. This does not affect its great biological interest due to the exceptional variety of polyphenol families and molecules of known beneficial activity on human health that can all be found in it (Table 2).

## 4. Structure and Function of Grape Polyphenol

Recently, the polyphenols intake has been estimated to be 1607 mg/d in a French well-balanced diet [81] which is above than previously found in epidemiological studies [116]. Grape is one of the major fruit crops produced worldwide and wine is the most widespread alcoholic beverage consumed. Moreover, the by-products of wine production are also a rich resource of biologically active molecules, which need to be emphasized since they possess the same phytochemicals of wine without the deleterious effect of alcohol [117].

Accumulating evidence shows that grape polyphenols regulate several mechanisms to prevent oxidative stress and inflammatory-mediated diseases [118] (Figure 1). Thanks to the phenolic groups, polyphenols can neutralize ROSP due to a transfer of electrons and/or hydrogen atoms to form phenoxyl radicals which are relatively more stable, thanks to resonance stabilization [42]. Polyphenols can also activate the endogenous antioxidant system via the ancestor Nrf2- Kelch-like ECH-associated protein 1 (KEAP1) signaling pathway [119,120]. Then, in aged muscle, grape polyphenols can increase the antioxidant enzymes such as catalase, SOD, glutathione reductase, and GPx leading to a reduction of muscle lipid damage associated with improvement in muscle function [121]. Resveratrol, and grape polyphenols, can increase the endurance capacity of muscle, which is related to increase reliance on mitochondrial lipid oxidation [107,122]. Polyphenols can increase mitochondria biogenesis and function due to their ability to activate sirtuin 1 (SIRT1) a class III histone deacetylase, NAD+-dependent (Figure 1). SIRT1 regulates several important processes such as gene expression, metabolism, and oxidative stress response via deacetylation of lysine groups [123]. More specifically, SIRT1 activates PGC-1α, mitochondrial transcription factor A (TFAM), nuclear receptor peroxisome proliferator-activated receptor (PPAR), and nuclear respiratory factor (NRF), all involved in mitochondria biogenesis and function. Moreover, SIRT1 not only modulates mitochondrial function but also regulates FOXO protein acetylation which in turn modulates manganese SOD and catalase expression increasing the defense against oxidative stress [124]. The energy sensor, AMPK, could also activate SIRT1 since it regulates the cellular levels of NAD+. Grape polyphenols can phosphorylate and activate AMPK [122]. Then, polyphenols can increase lipid oxidation since it is well known that AMPK controls malonylCoA level a major inhibitor of the CPT and lipid entry into mitochondria [125] but also AMPK controls mitochondrial biogenesis via the deacetylation and activation of PGC-1α by SIRT1 [107]. Indeed, grape polyphenols supplementation increases muscle PGC-1α alpha mRNA and maintains CPT1 mRNA after fructose ingestion in first-degree relatives of T2D patients, suggesting a conserved lipid oxidation capacity which could explain the body weight gain decrease in this group compared to placebo [126]. The inflammation pathway is also negatively regulated by SIRT1 through NFĸB acetylation explaining in part the anti-inflammatory activity of polyphenols. On the other part, resveratrol has been found to alleviate obesity-induced skeletal muscle inflammation due to a shift in macrophage toward an anti-inflammatory profile and a decrease in TLR4 expression [127]. In the context of muscle atrophy, resveratrol has demonstrated an ability to decrease atrogin-1 and MuRF-1 expression depending on SIRT1 activity [128]. Then, polyphenols but mostly resveratrol have been described as exercise mimetic compounds according to their positive regulation of muscle mass [129]. Polyphenols are secondary metabolites of vegetables and fruits (largely in berries) with important physiological and defense functions as pigments and antioxidant molecules. They are accumulated in the berries, especially in the solid parts (mainly skin and seeds), in response to various internal and external stimuli (growth, free radicals excess, ultraviolet (UV) radiations, fungi, insects, and bacteria attack) [130]. Then, polyphenols content in grapes is highly variable depending on the cultivar, maturity, and fermentation processes during wine production [131]. They play an important role in maintaining the organoleptic characteristics of wines (color, taste, and astringency), and thanks to their antimicrobial activity they are used as preservatives in the food and cosmetic industries [132,133]. By highlighting their antioxidant properties [134,135], the French paradox stimulated the exploration of the effects that polyphenols from different sources could have on various diseases [136]. As we stated at the outset, the study of grape polyphenols on muscle is still in its infancy, and therefore studies specifically demonstrating the effects of polyphenols extracts obtained from grapes are still scarce. That is why in this section, we will report studies on main polyphenols (individual molecules or groups of molecules) found in grape, that can be also isolated from other sources [137]. In fact, even if isolated from other sources, these polyphenols show the same chemical structure, and we reasonably assume that they could exert the same mechanism of action. For each subfamily, we will cite studies focusing on their effects on obesity, aging, T2D, inflammation, and oxidative stress, with a particular look at pathways affecting muscle. The chemical structure of polyphenols is characterized by the presence of one or more hydroxyl groups and one or more aromatic rings with six carbon atoms. 

**Figure 2:**
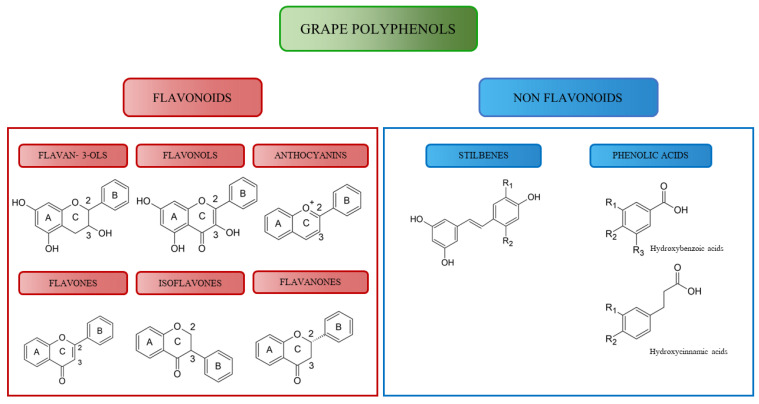
Polyphenols classification.

Polyphenols are a wide class of compounds with more than 8000 molecules isolated and described [138]. Thus, for easier understanding, polyphenols are divided into two big families according to the number of aromatic rings, the way they are linked to each other, and the position and oxidation state of the hydroxyl groups: flavonoids and non-flavonoids, each of which contains other classes (Figure 2). 

### 4.1. The Flavonoids

Flavonoids are known as plant pigments but also for their antioxidant, antimicrobial, and light-screening functions [139]. Moreover, thanks to their properties they are successfully used in the pharmaceutical and food sectors as preservatives and pigments [140]. Depending on the chemical structure, oxidation degree, or unsaturation of the heterocyclic ring C, flavonoids can be further classified into six mean groups, notably: flavanols, flavonols, anthocyanes, flavones, isoflavones, flavanones [141]. These molecules are generally water-soluble, and they can be found in glycosylated or aglycone form. Their basic structure is the flavone ring. 

#### 4.1.1. Flavan-3-ols

Flavanols are relevant molecules in grapes and wine for their contribution to color stabilization and their astringent and bitter properties [142]. They are generally referred to as catechins and in their structure, the double bond on the C ring is absent and, accordingly, two chiral carbons are found (C2 and C3, see Figure 2) [143]. The monomeric catechins can exist in the form of four stereoisomers, depending on the hydroxylation on the C4 of the C ring. The *trans* isomer is called catechin, the *cis* one is called epicatechin. When catechins and epicatechins polymerize, they form condensed tannins also known as proanthocyanidins [144]. The organoleptic and pharmacological properties of tannins are strictly related to their structure and polymerization degree. Molecules with a higher polymerization degree have stronger radical scavenger activity and moreover, they are more bioavailable thanks to increased resistance to acid hydrolysis in the stomach [145]. Indeed, grape seed proanthocyanidins have been found to promote health-benefit via inhibition of protein damage linked to their antioxidant capacities and enzyme inhibition [146]. Grape seed proanthocyanidins extract is able to activate AMPK in C2C12 myotubes, consequently switching on Nrf1, SIRT1, and PGC-1α. This mechanism is behind the increase in slow myosin heavy chain, decrease in fast myosin chains, increased activity of succinic dehydrogenase and malate dehydrogenase, and decreased activity of lactate dehydrogenase all of these leading to increased resistance to fatigue [91]. (-)-Epigallocatechin-3-gallate (EGCG) wields an antioxidant activity through a combination of mechanisms. On one side it acts as a radical scavenger or by chelating metal ions that favor the formation of radicals [85], on the other, it enhances the activity of endogenous antioxidant enzymes as well as inhibits the activity of pro-oxidant enzymes and pro- inflammatory TLR4 pathways [57,86,87]. EGCG has a role in mitigating the oxidative stress-based inflammation in T2D and obesity by regulating the NFĸB, Adenosine Monophosphate Activated Protein Kinase (AMPK), and Mitogen-Activated Protein Kinases (MAPK) signaling pathways, thus decreasing IR and increasing muscle lipid oxidation [88,89]. EGCG has a beneficial effect on the aging process by promoting autophagy through activation of Beclin 1 and apoptosis by induction of caspase proteins [90], see Table 2.

#### 4.1.2. Flavonols 

The flavonols exhibit a double bond between the carbons C2 and C3 and the C3 is hydroxylated. In this position, the aglycones can be linked to different sugars (often glucose and rhamnose) [147]. They are found in big amounts in the grape skin because their biosynthesis is stimulated by the sun light, from which they protect the plant. Quercetin, one of the major flavonoids in grapes, reduces inflammatory signaling pathway by inhibiting inflammatory receptors in mice with obesity-induced skeletal muscle atrophy [148] and attenuates adipogenesis and fibrosis in a human muscle-derived mesenchymal progenitors cells model [149]. In human myotubes from a healthy donor, at a physiological dose, quercetin modestly increases the insulin signaling pathway and glycogen storage which could participate in improving insulin sensitivity [150]. Their signaling pathway expresses an anti-inflammatory activity through the activation of Nrf2/Antioxidant Responsive Element (ARE) pathways with a subsequent upregulated synthesis of antioxidant endogenous enzymes [92]. Furthermore, it was described that quercetin reduces inflammation in the adipocytes and macrophages by reducing the expressions of genes encoding for TNF-α, IL-6, IL-1β, Cyclooxygenase-2 (COX-2), Inducible Nitric Oxide Synthase (iNOS) and also by keeping away from the activation of NFĸB [93]. Myricetin, another of the flavonoids most present in red grapes, has been shown to have anti-diabetic effects in numerous studies [94]. On C2C12 myotubes, it was demonstrated that myricetin increases glucose uptake thanks to the activation of AMPK and Akt signaling pathways, thus decreasing insulin resistance [151]. Kaempferol, as well, is known to be an anti-inflammatory compound. The different mechanisms were summarized by Alam et al. in 2020 and they include: decreased release of IL-6, IL-1β, 18, and TNF-α, activation of the Nrf2 pathway and synthesis of target enzymes, and inhibition of TLR4 [95]. 

#### 4.1.3. Anthocyanes

The anthocyanes (or anthocyanins) are, with the chlorophyll and the carotenoids, the most important vegetal pigments [152]. In grapes, anthocyanins are located in the skin and exhibit a strong antioxidant power, to protect the plant from the damage caused by UV radiation [153]. The base structure is the 3,5,7,4’-tetrahydroxyflavylium cation or flavylium cation. The OH group in position 3 is always glycosylated and the one in position 5 is very frequently. There are many studies that show the beneficial antioxidant and anti-inflammatory effects of anthocyanin supplementation on obesity state meticulously reported by Sivamaruthi et al. [97]. To cite one, high fat diet obese mice supplemented with 250 mg/kg/d grape pomace extract, rich in anthocyanin, decreased the levels of plasma C-reactive protein after 12 weeks, thus exerting an anti-inflammatory activity [154]. Many studies have already described their disparate biological activities. Among them, anthocyanins have been shown to exhibit an inhibitory effect on the enzymes COX-1 and COX-2, thus reducing systematic and cardiovascular inflammation [96]. Moreover, glucose and lipid metabolism are also improved following anthocyanin supplementation. For example, cyanidin-3-*O*-glucoside supplementation increases the expression of Peroxisome Proliferator-Activated Receptors (PPARs), thus reducing dyslipidemia and increasing sensitivity to insulin in mice after eight weeks of supplementation by increasing lipid oxidation [98] and plays a role to retrieve IR in diabetes by re-establishing insulin secretion and decreasing IL-1β and IL-6 concentrations [99]. 

#### 4.1.4. Flavones

These molecules, vegetal yellow pigments, have the fundamental skeleton of the flavone (also known as 2-phenylchromone), with the presence of a double bond between C2 and C3 and no hydroxyl group in C3. It is therefore an oxidized form of flavanones. Like other flavonoids, they mostly appear in the form of water-soluble glycosides. Luteolin and apigenin showed in lipopolysaccharide (LPS)-activated macrophages a strong anti-inflammatory effect thanks to the decreased production of NO and prostaglandin E2 [100]. It was also described as a beneficial effect of apigenin in mitigating obesity-induced atrophy in mice. After the treatment, the size of muscle fibers was enhanced, and mitochondria were increased in number and volume. The effect has been scribed to the counteracting of oxidative species and improving the activity of antioxidant enzymes such as SOD and GPx [101]. 

#### 4.1.5. Isoflavones 

Isoflavones, commonly known as phytoestrogens, are isomers of flavones in which the phenyl group (ring B) is linked to the C3 and not to the C2 of ring C. They are synthetized following a microbial attack or in stress conditions, thus acting as phytoalexins [155]. It described their anti-inflammatory activity and their effects on the mitigation of T2D. Daidzein acts as an inhibitor of the enzymes α-glucosidase and α-amylase, thus decreasing post-prandial glycemia [102]. Additionally, daidzein acts on different targets involved in the insulin response (AMPK, Glycerol Kinase (GK), Glucose 6-Phosphatase (G6Pase), Phosphoenolpyruvate Carboxykinase (PEPCK), PPARγ, Glucose Transporter 4 (GLUT4), Insulin Receptor Substrate 1 (IRS1), IRS2, etc.) and in the anti-inflammatory response (PPARγ, TNFα, NFĸβ, IL-6, Chemokine ligand 2 (Ccl2), Chemokine (C-X-C motif) ligand 2 (Cxcl2), etc.) [103]. In obese patients, genistein (50 mg/day for 2 months) ameliorates IR associated with an increase in skeletal muscle AMPK activation, thus increasing fatty acid oxidation and insulin sensitivity [104]. 

#### 4.1.6. Flavanones

All the molecules of this class have a structure based on the progenitor flavone, with a double bond between C2 and C3. We can find flavanones in grapes and some representative molecules such as naringenin and hesperetin which exhibit important anti-inflammatory, antioxidant, and antidiabetic action. In fact, naringenin is able to ameliorate hyperglycemia and to improve the secretion of insulin. Moreover, it has an action on the inflammatory status by decreasing cytokines like TNFα and IL-6 and increasing the activity of SOD [105]. Naringenin and hesperetin are aglycones, but they are often found in their glycosylated form called naringin (naringenin + neohexperidose) and hesperidin (hesperetin + rutinose). It was demonstrated that hesperidin has an antidiabetic action, exerted by upregulating IRS, Akt, and GLUT4 in muscle cells, which is higher than the aglycone hesperetin [106]. 

### 4.2. Non-Flavonoids

#### 4.2.1. Stilbenes

Stilbenes are known as phytoalexins, protective compounds secreted by the plant following contact with a pathogen or an abiotic stress [156]. Stilbenes are diaryl ethers, ethenes substituted with a phenyl group on both carbon atoms of the double bond. Thus, there are two possible geometric isomers of stilbene, *trans* and *cis* [157]. Grape berries are an excellent source of trans-resveratrol, the most notable compound among stilbenes, with a concentration of 10–100 higher than in other berries [158]. Trans-resveratrol is the molecule belonging to this class that has been most investigated for its biological properties [159] due to the activation of Sirtuin 1 (SIRT1). Lagouge et al., in a groundbreaking paper, showed that resveratrol activates the deacetylase SIRT1 and the coactivator Peroxisome Proliferator-Activated Receptor-Gamma Coactivator-1α (PGC-1α), thus increasing mitochondrial activity, its decrease is a cause of aging and metabolic diseases, and miming caloric restriction and exercise [107]. Then, resveratrol can be used successfully against different pathologies [160]. Another beneficial effect on metabolism is given by the fact that resveratrol improves insulin sensitivity by activating Akt, and AMPK pathways and inhibiting NFkB favoring insulin signaling, lipid oxidation, and decreasing inflammation in rodents [107,108]. Besides its beneficial metabolic effect, resveratrol has been efficient in animal models or cell cultures for preventing muscle atrophy due to dexamethasone [128], mechanical unloading [129], cancer cachexia [161], and sarcopenia in obese rodents [109,110]. Interestingly, resveratrol has been shown to decrease oxidative stress and inflammation associated with aging without a reversal of muscle atrophy [162,163].

#### 4.2.2. Phenolic Acids

Phenolic acids are one of the classes of phenolic compounds found in higher concentrations in the plant world [164]. This class of compounds is divided into hydroxybenzoic acids and hydroxycinnamic acids, in the function of the position of the carboxylic group on the aromatic ring (C1-C6 or C3-C6 structure) [143]. They are often found in conjugated form with tartaric acid or glucose, forming soluble compounds [165]. Phenolic acids are known to have a beneficial impact on human health, acting as oxidative species scavengers but also regulating some key signaling pathways. As an example, phenolic acids exert an adjuvant effect on diabetes by activating the Phosphatidylinositol 3-kinase (PI3K)/Akt pathway, increasing the translocation of GLUT4 in adipose and muscle tissues and thus insulin sensitivity [111]. Gallic acid and *p*-coumaric acid showed to have a potent hypoglycemic and lipid-lowering effect on diabetic rats, exerted by decreasing TNF-α and modulating PPAR-γ in adipose tissue [112] and by modulating muscle AMPK [166]. Caffeic acid phenetyl ester, a derivative of caffeic acid found in grapes, inhibits the enzymes COX and lipoxygenase (LOX), the main enzymes involved in inflammation [113]. To bring some examples of biological activities among the hydroxybenzoic acids, vanillic acid can be successfully employed in obesity because it decreases the adipogenic PPAR and CCAAT-enhancer-binding proteins α (C/EBPα) and increases lipid oxidation through AMPKα [114]. Syringic acid is an antidiabetic agent, which combines an antihyperglycemic effect with the mitigation of diabetic neuropathy. The effect is given by the activation of PGC-1α and Nrf1, with consequent increases in mitochondrial biogenesis, and decreased secretion of pro-inflammatory cytokines (IL-6, IL-1β, and TNF-α) [115]. 

It is evident nowadays that introducing dietary grape polyphenols through alimentation is essential for maintaining a good state of health. Several in vitro and in vivo studies and clinical trials demonstrated that their action on the body is exerted by regulating metabolism, weight, and muscle function, mitigating oxidative stress, inflammation, and chronic diseases [122,126,167]. 

Although their antioxidant action is considered their main mechanism of action, this action alone is not able to explain all the biological effects of grape polyphenols. In fact, it has been proven that they also act through the modulation of receptors [168], transcription factors during myogenesis [169,170], enzymes activities [171,172], and also through epigenetic modulation [173], and non-coding (nc) RNA regulation [174].

## 5. Metabolism of Polyphenols

Structures and activities of polyphenols could be altered by their interaction with other molecules contained within the food matrix and of course by hepatic and intestinal metabolism. Consequently, human plasma concentrations of polyphenols are not comparable to those concentrations described as necessary to achieve a great biological activity as demonstrated by in vitro studies. Their metabolism and bioavailability vary considerably from molecule to molecule and there is a strong hypothesis that polyphenols’ metabolites produced in vivo are also responsible for the biological action of polyphenols [175].

The series of processes involved in the metabolization, and absorption of polyphenols begins in the oral cavity with saliva and then continues in the gastrointestinal tract involving the gut microbiome. After these processes, a part of the polyphenols will be absorbed in their original form, a part will be excreted in the feces and a part will be transformed into new molecules with biological effects. The destiny of polyphenols in the digestive system depends on their original chemical structure: different polyphenols will undergo different transformations. 

Saliva is composed mainly of water, salts, enzymes, and proteins (albumin, α-amylase, sulphomucins, sialomucins, glycoproteins, sulfated cystatins, agglutinins, histatins, lysozymes, mucins, immunoglobulins, proline-rich proteins) among which amylase is the most abundant [176]. In the mouth, through mastication polyphenols are mixed with saliva and solubilized. Molecules such as tannins can form a complex and precipitate with the tannin-binding salivary proteins (TBSPs). These complexes remain stable during the transit in the stomach, while they are solubilized in the intestine in presence of bile salts [177]. Lipophilic polyphenols such as resveratrol, curcumin, and quercetin are poorly bioavailable because of their lack of solubility, thus limiting their antioxidant action in the body. Saliva has been described as able to solubilize lipophilic polyphenols, thus increasing their bioavailability and their antioxidant activity [178]. 

After the mechanical and chemical transformations in the mouth, polyphenols are transported to the gastrointestinal tract. Absorption can occur by passive transport or, much more frequently because of lipophilia, by carrier-mediated active transport. In rats, phenolic acids [179] and not glycosylated flavonoids [180] can be absorbed at the stomach level. Chen et al. described that among the total polyphenolic compounds only 5–10% can be directly absorbed in the small intestine while the rest must undergo transformations by enzymes in subsequent sections of the gastrointestinal tract before they can be absorbed [179]. For instance, glycosylated flavonoids, such as quercetin, are poorly absorbed due to their hydrophilic character. They must be deglycosylated by β-glucosidases of the small intestine and then absorbed as aglycones [181]. The human digestive tube is populated by a copious microbial population, counting more than 100 trillion different microorganisms, whose name is gut microbiota. Gut microbiota has a big impact on the polyphenols’ absorption and bioavailability, because of their own enzymatic capacities. First, the *O*-glycosides are hydrolyzed to aglycones, which will further undergo reactions of glucuronidation or sulfonation [182]. For example, trans-piceid, which is chemically an *O*-glycoside of resveratrol, is hydrolyzed to free resveratrol by the gut microbiota [183]. Subsequently, both molecules are largely sulfonated or glucuronidated but also hydroxylated, to produce different derivatives such as dihydroresveratrol, dihydropiceid, and many others [184]. Moreover, the gut microbiota is able to perform catabolic reactions, like degradation of aromatic rings via carbon-carbon cleavage, decarboxylation, hydrogenation, ihydroxylation, demethylation, thus forming derivatives with simpler structures [182]. Cyanidin, taken as a model of anthocyanidin, is metabolized by the gut microbiota starting by the opening of the pyranic ring followed by a second carbon-carbon cleavage, giving protocatechuic acid and 2-(2,4,6-trihydroxyphenyl) acetic acid as final products [185]. Hydrolysable tannins are complex phenolic compounds, metabolized by the gut microbiota. The first enzymes involved are hydrolases (tannin acyl hydrolase) which release gallic acid (gallotannins) or ellagic acid (ellagitannins). Gallic acid is further transformed by decarboxylation and hydroxylation, while ellagic acid only transforms by dihydroxylation [186].

Ferulic acid is mostly found in its esterified form. Its methyl ester, for example, is readily demethylated to ferulic acid then its double bond is saturated by hydrogenation, the methoxy group on the carbon 3 is demethylated and the carbon 4 is dehydroxylated to obtain *3*-phenylpropionic acid [187]. The polyphenols’ metabolites thus formed will be partly absorbed into the systemic circulation and partly excreted as waste to terminate their biological activity. Apart from the metabolization carried out by the gut microbiota, they can enter the enterohepatic cycle and undergo phase I and phase II metabolism, eventually going back from the liver to the intestine through the bile [188]. Phase I metabolism is carried out in the liver by the cytochrome P450 (CYP450) superfamily of enzymes, and it involves reactions of oxidation, hydrolysis, and reduction. Phase II metabolism has the aim to conjugate polyphenols to augment their hydrophilicity and help their rapid elimination from the body. Phase II enzymes include UDP-glucuronosyltransferase, responsible for glucuronidation, N-acetyltransferase, which catalyzes the transfer of acetyl groups from acetyl-CoA to polyphenols, glutathione-S-transferase which leads the polyphenol to conjugation with a reduced glutathione molecule [189]. It was reported for instance, that naringin and naringenin are susceptible to phase I and phase II metabolism. In fact, they are firstly oxidized or demethylated by CYP450 and subsequently glucuronidated, sulfated and methylated. From the metabolism of the only naringin 32 metabolites are derived, some keeping the flavonoid structure and some only a phenolic one [190]. As another example, it is possible to find in the human intestine quercetin-3′-*O*-glucuronide and quercetin-4′-*O*-glucuronide as a result of phase II metabolism on quercetin [191]. Another well-studied molecule is resveratrol, which when it reaches the human gastrointestinal tract goes through reactions of sulfation and glucuronidation, phase II reactions, generating a variety of reported metabolites, such as trans-resveratrol-3-*O*-sulfate, trans-resveratrol-4′-*O*-sulfate, *trans*-resveratrol-3,4′-disulfate, *trans*-resveratrol-3-*O*-glucuronide and *trans*-resveratrol-4′-*O*-glucuronide [192].

## 6. Clinical Studies 

The number of clinical studies evaluating skeletal muscle mass and function for patients with chronic/non-communicable diseases and following grape polyphenols administration is strongly limited compared to in vitro and animal studies. This relative absence of clinical trials is particularly difficult to understand considering the encouraging results obtained in vitro, in animal models, and during the scarce clinical studies yet realized some years ago [126,193,194,195]. In 2011, Brasnyo et al. studied the effect of supplementation of resveratrol (10 mg/d) in a group of 10 T2D subjects for four weeks [193]. This low dose of resveratrol improved insulin sensitivity, decreased blood glucose levels, decreased blood oxidative stress, and increased Akt phosphorylation in platelets without any data on muscles. The same year, Timmers et al. demonstrated the beneficial effect of nutritional supplementation of 150 mg/d of resveratrol for 30 d in 11 healthy obese men [194]. This supplementation clearly induced metabolic changes in obese humans, mimicking the effects of calorie restriction or endurance exercise. In fact, Timmers observed activation of AMPK in muscle biopsies of the resveratrol-treated subjects, increased PGC-1α and citrate synthase activity, attesting to an increased muscle mitochondrial activity. Interestingly, resveratrol decreased hepatic lipid content but increased intra-myocellular lipid content. Hokayem et al. showed that eight weeks of supplementation with a natural mixture of grape polyphenols at nutritional doses (2 g/d), efficiently prevents fructose-induced oxidative stress and IR in healthy overweight/obese first-degree relatives of T2D patients [126]. At the skeletal muscle level, grape polyphenols supplementation protected mitochondrial function, prevented oxidative stress, and tended to increase insulin sensitivity after fructose challenge Goh et al. showed that supplementation with 3 g resveratrol daily for 12 weeks regulates energy expenditure through increased skeletal muscle SIRT1 and AMPK protein expression in patients with T2D [195]. These interesting results indicate that resveratrol may have beneficial exercise-mimetic effects in patients with T2D [195]. However, no significant modification in systemic insulin sensitivity was observed during this study whereas a decrease in glycated hemoglobin was observed, suggesting an improvement in glucose tolerance. The number of volunteers included in this trial, 10 subjects, could be not enough to have a clear demonstration of grape polyphenols’ effects on systemic insulin sensitivity. In seventeen well-controlled T2D subjects, supplementation of 150 mg/d of resveratrol for 30 d was also not able to change insulin sensitivity whereas it increased lipid-derivate mitochondrial respiration in muscle [196]. An 8-week supplementation of obese subjects with red wine polyphenols (600 mg/day) does not also improve obesity-associated IR [197]. The main limitation of this study was that total polyphenols intake was similar between the treated and placebo groups limiting the conclusions on the supplementation. Overall, the improvement of insulin sensitivity with grape polyphenols (resveratrol or polyphenols mixture) was mainly found in metabolically stressed patients at a systemic level but not in a normal state of health [198], whereas the improvement in mitochondrial function and oxidative stress were evident whatever the tissues and the metabolic status of patients. These data underline that in obese/T2D humans, grape polyphenols seem to act mainly as antioxidants. 

Clinical trials for investigating polyphenols supplementation on aged muscle are even more limited. In an attempt to decrease functional limitations, most studies on elderly subjects have combined exercise training and polyphenols supplementation in order to amplify the benefits of exercise alone. One study found the beneficial effect of 500 mg/d of resveratrol supplementation associated with exercise on muscle force with higher mitochondrial density and muscle fatigue resistance in elderly subjects allowing to potentially reverse sarcopenia [199]. However, most of the studies report no effect of the combination [200] or even detrimental effect on muscle [201,202]. Recently, in a pilot study on elderly community-dwelling adults, Harper et al. found that 1000 mg/d of resveratrol permits a 33.1 m improvement in the 6-min walk distance associated with higher mitochondrial function and decreased inflammation [203]. This data could be related to the epidemiologic studies where an association between the Mediterranean diet with gait speed is found [204]. However, the beneficial effect of grape polyphenols supplementation on sarcopenia needs to be investigated. To date, no prospective study with data on sarcopenic or frailty subjects is available but some clinical trials are ongoing [124].

## 7. Conclusions

Restoration of skeletal muscle mass and function is vital to cure the comorbidities associated with obesity and aging. Grape polyphenols have therapeutic potential for such diseases mediated by oxidative stress and inflammation. Nevertheless, large-scale clinical trials are still necessary to better investigate the activity of these natural compounds at the skeletal muscle level. Moreover, the majority of clinical trials have studied the effect of one purified polyphenol, resveratrol, and not the activity of a crude extract or of a mixture of several molecules of polyphenols. Yet in vitro studies or animal models, even not concerning skeletal muscle, showed an interest in using a mixture of polyphenols. In fact, such a mixture of polyphenols is more active than each individual molecule alone as a synergy effect occurs between the molecules [205,206]. 

Another important point in using a mixture of polyphenols is that an extract in which many polyphenolic molecules are blended together in low concentrations has the advantage of reducing the toxicity that could be derived from the use of a single molecule in higher concentrations [207].

Additionally, the advantage of grapes over other sources of polyphenols is that grapes, after strawberries and lychees, are among the fruits with the highest polyphenols content (anthocyanins/anthocyanidins, flavonols) [208]. As shown in Table 1, moreover grape boasts a wide variety of phenolic molecules. Although stilbenes, such as resveratrol, have a lower relative abundance than the previously mentioned molecules in grapes, it is at least 10–100 times higher than in other berries [209], making grapes one of its primary sources. Grapes are consumed worldwide. They are the fourth most abundant fruit crop cultivated all over the world [210], so their supply as a source of extraction is easy and affordable for many countries. Moreover, there is an environmental and eco-friendly aspect to using grape polyphenols. Indeed, grape skins and grape seeds are waste by-products of wine or grape juice production. They are rich sources of polyphenols and could be used in the form of food supplements. 

Translational data confirming the benefit of grape polyphenols in human health at the muscle level would allow the development of grape polyphenols supplementation in therapy and in the management of obese and aging people on a routine basis. This would improve the quality of life and decrease the economic cost of medical care for obese and elderly patients.

## Figures and Tables

**Table 1:** Polyphenols characterization, total polyphenol content (TPC) of major well-known vegetal sources of dietary polyphenols. TPC is expressed in mg/100 g of fresh weight (FW). Data in the table were extracted from the PhenolExplorer database [82,83,84].

Source	Family of Polyphenol	Amount	Mean TPC (Folin Assay)
Apple	Anthocyanins	0.93 mg/100 g FW	200.96 mg/100 g FW
	Dihydrochalcones	5.38 mg/100 g FW	
	Flavanols	24.12 mg/100 g FW	
	Flavonols	6.86 mg/100 g FW	
	Phenolic acids	19 mg/100 g FW	
Artichoke, heads, raw	Flavones	57.8 mg/100 g FW	1142.40 mg/100 g FW
	Phenolic acids	202.23 mg/100 g FW	
Blueberries	Flavonols	12.23 mg/100 g FW	151.33 mg/100 g FW
	Phenolic acids	162.47 mg/100 g FW	
	Phenolic acids	37.06 mg/100 g FW	
	Other polyphenols	0.45527 mg/100 g FW	
Cocoa, powder	Flavanols	511.62 mg/100 g FW	5624.23 mg/100 g FW
	Phenolic acids	37.06 mg/100 g FW	
	Other polyphenols	0.45527 mg/100 g FW	
Grape	Anthocyanins	72.1 mg/100 g FW	184.97 mg/100 g FW
	Flavanols	17.11 mg/100 g FW	
	Flavonols	3.08 mg/100 g FW	
	Phenolic acids	1.69 mg/100 g FW	
	Stilbenes	0.3362 mg/100 g FW	
Green tea	Flavanols	71.18 mg/100 g FW	61.86 mg/100 ml
	Flavonols	5.29 mg/100 g FW	
	Phenolic acids	12.53 mg/100 g FW	
Olives, green	Flavones	0.56 mg/100 g FW	161.24 mg/100 g FW
	Phenolic acids	134.94 mg/100 g FW	
	Other polyphenols	211.05 mg/100 g FW	
Persil, fresh	Other polyphenols	13.95 mg/100 g FW	89.27 mg/100 g FW
Strawberries	Anthocyanins	73.01 mg/100 g FW	289.20 mg/100 g FW
	Flavanols	9.1375 mg/100 g FW	
	Flavonols	2.32 mg/100 g FW	
	Phenolic acids	10.74 mg/100 g FW	
	Stilbenes	0.35 mg/100 g FW	

**Table 2:** Main effects of polyphenols present in grapes on mechanisms involved in obesity and aging.

Family and Subfamily	Compound	Effect and Mechanism	References
**Flavonoids/Flavan-3-ols**	*EGCG*	● **Antioxidant** Radical scavenging Metal ion chelation ↑ CAT, ↑ SOD1 e SOD2, ↑ GPx ● **Anti-inflammatory** ↓ NFĸB via ↓ Iĸβ ↓ COX-2 ↓ IRF3 via ↓ TBK1 ● **Anti-diabetic** ↓ insulin resistance ↑ lipid oxidation in muscle ↑ NFĸB, ↑ AMPK, ↑ MAPK ● **Anti-aging/**pro-apoptotic ↑ Beclin-1 and ↑ caspases	● Fraga et al. [85] Bernatoniene et al. [86] Meng et al. [57] ● Youn et al. [87] ● Casanova et al. [88] Li et al. [89] ● Pallauf and Rimbach [90]
	Grape seed proanthocyanidins	● **Anti-diabetic** ↑ Nrf1, ↑SIRT1, and ↑PGC-1α, ↑ slow myosin heavy chain, ↑ succinic dehydrogenase and malate dehydrogenase activities, ↑ resistance to fatigue	● Xu et al. [91]
**Flavonoids/Flavanols**	*Quercetin*	● **Anti-inflammatory** ↑ Nrf2/ARE pathways ↑ Antioxidant enzymes ↓ TNF-α, ↓ IL-6, ↓ IL-1β, ↓ COX-2, ↓ iNOS, ↓ NFĸB in adipocytes and macrophages	● Costa et al. [92] ● Sato et al. [93]
	Myricetin	● **Anti-diabetic** ↑ glucose uptake, ↓ insulin resistance, ↑Akt and ↑AMPK signaling pathways	● Pandey et al. [94]
	*Kaempferol*	● **Anti-inflammatory** ↓ IL-6, IL-1β, 18 and TNF-α ↑ Nrf2 and synthesis targets Inhibition TLR4	● Alam et al. [95]
**Flavonoids/Anthocyanes**	*Anthocyanins*	● **Anti-inflammatory** ↓ COX-1 and COX-2 ↓ C-reactive protein	● Mozos et al. [96] ● Sivamaruthi et al. [97]
	*Cyanidin-3-O-glucoside*	● **Antidyslipidemic** ↑ PPARs ● **Anti-diabetic** ↑ Insulin sensitivity → ↑ PPARs ↑ Insulin secretion → ↓ IL-1β and IL-6 ↑ TLR4/IĸBα pathway	● Jia et al. [98] ● Geng et al. [99]
**Flavonoids/Flavones**	*Luteolin/Apigenin*	● **Anti-inflammatory** ↓ NO and ↓ PGE2	● Tian et al. [100]
	*Apigenin*	● **Anti-obesity** Radical scavenger ↑ Increase muscle fibers size ↑ number and volume mitochondria ↑ SOD and GPx	● Wang et al. [101]
**Flavonoids/Isoflavones**	*Daidzein*	● **Anti-diabetic** Inhibition α-amylase and α-glycosidase ↑ AMPK, ↑ GK, ↓ G6Pase, ↓ PEPCK, ↑ GLUT4, ↑ IRS1, ↑ IRS2, ↑ PPARγ ● **Anti-inflammatory** ↑ PPARγ, ↓ TNFα, ↓ NFĸB, ↓ IL-6, ↓ Ccl2, ↓ Cxcl2	● Park et al. [102] ● Das et al. [103]
	*Genistein*	● **Anti-diabetic** ↑ AMPK in skeletal muscle ↑ insulin sensitivity ↑ lipid oxidation	● Guevara-Cruz et al. [104]
**Flavonoids/Flavanones**	*Naringenin*	● **Anti-diabetic** ↑ Insulin secretion ● **Anti-inflammatory** ↓ TNF-α and IL-6 ↑ SOD	● Rehman et al. [105]
	*Hesperidin*	● **Anti-diabetic** ↑ IRS, ↑ Akt, and ↑ GLUT4 in muscle cells	● Dhanya et al. [106]
**Stilbenes**	*Resveratrol*	● **Anti-diabetic** ↑ SIRT1 and ↑ PGC-1α ↑ mitochondrial activity (exercise mimetic effect) ↑ Akt and AMPK pathways → ↑ insulin sensitivity ● **Anti-obesity** ↓ fat accumulation ↑ lipolysis ● **Anti-aging** ↓ caspase 3	● Lagouge et al. [107] ● Lagouge et al. [107] Kang et al. [108] ● Huang et al. [109] ● Bai et al. [110]
**Phenolic acids**	*Phenolic acids*	● **Anti-diabetic** ↑ GLUT2 in pancreatic β-cells ↑ PI3K/Akt and ↑ GLUT4 in adipose and muscle tissues ↓ α-glucosidase activity	● Kumar et al. [111] ● Duboit et al. [96]
	*Gallic acid/p-coumaric acid*	● **Anti-diabetic and anti-obesity** ↓ TNF-α and ↓ PPAR γ in adipose tissue	● Abdel-Moneim et al. [112]
	*Caffeic acid phenetyl ester*	● **Anti-inflammatory** ↓ COX and ↓ LOX Inhibition detachment *arachidonic acid.*	● Silva et al. [113]
	*Vanillic acid*	● **Anti-obesity** ↓ PPAR and C/EBPα ↑ Lipid oxidation through ↑ AMPKα	● Jung et al. [114]
	***Syringic acid***	● **Anti-diabetic** ↑ PGC-1α and Nrf2 ↑ increased mitochondrial biogenesis. ↓ TNF-α, IL-1β, and IL-6	● Rashedinina et al. [115]
